# High-Throughput Switchgrass Phenotyping and Biomass Modeling by UAV

**DOI:** 10.3389/fpls.2020.574073

**Published:** 2020-10-20

**Authors:** Fei Li, Cristiano Piasecki, Reginald J. Millwood, Benjamin Wolfe, Mitra Mazarei, C. Neal Stewart

**Affiliations:** ^1^Department of Plant Sciences, University of Tennessee, Knoxville, Knoxville, TN, United States; ^2^Center for Bioenergy Innovation, Oak Ridge National Laboratory, Oak Ridge, TN, United States

**Keywords:** phenotype, LiDAR, spectral index, biomass, Nitrogen

## Abstract

Unmanned aerial vehicle (UAV) technology is an emerging powerful approach for high-throughput plant phenotyping field-grown crops. Switchgrass (*Panicum virgatum* L.) is a lignocellulosic bioenergy crop for which studies on yield, sustainability, and biofuel traits are performed. In this study, we exploited UAV-based imagery (LiDAR and multispectral approaches) to measure plant height, perimeter, and biomass yield in field-grown switchgrass in order to make predictions on bioenergy traits. Manual ground truth measurements validated the automated UAV results. We found UAV-based plant height and perimeter measurements were highly correlated and consistent with the manual measurements (*r* = 0.93, *p* < 0.001). Furthermore, we found that phenotyping parameters can significantly improve the natural saturation of the spectral index of the optical image for detecting high-density plantings. Combining plant canopy height (CH) and canopy perimeter (CP) parameters with spectral index (SI), we developed a robust and standardized biomass yield model [biomass = (*m* × SI) × CP × CH] where the *m* is an SI-sensitive coefficient linearly varying with the plant phenological changing stage. The biomass yield estimates obtained from this model were strongly correlated with manual measurements (*r* = 0.90, *p* < 0.001). Taking together, our results provide insights into the capacity of UAV-based remote sensing for switchgrass high-throughput phenotyping in the field, which will be useful for breeding and cultivar development.

## Introduction

Switchgrass (*Panicum virgatum* L.) is a native North America prairie grass that has been studied as a potential bioenergy crop in the United States and Europe since the mid-1980s (Lewandowski et al., 2003). It is a perennial grass, with C_4_ metabolism, which is adapted to cultivation in much of the eastern United States and similar regions requiring low agronomic inputs (Vogel, 2004; Bouton, 2007; Schmer et al., 2008). It grows as a “clonal modular plant” from tillers (Boe and Casler, 2005). Each plant produces a population of tillers that can grow up to 4 m tall (Bouton, 2007). Switchgrass is highly self-incompatible, and its reproductive structures consist of a diffuse panicle arranged at the end of long branches (Barnes et al., 1995; Vogel, 2004). It produces high aboveground biomass each growing season as well as high lignin and cellulose content in cell walls (Vogel, 2004). The biomass produced by switchgrass serves as a feedstock for bioenergy production as an effort to create green energy to reduce the consumption of fossil fuels (McLaren, 2005; Naik et al., 2010).

Since the beginning of switchgrass bioenergy feedstock development, breeding programs have utilized germplasm with desirable phenotypes such as high biomass production, nutrient use efficiency and stress tolerance (Barney et al., 2009; Jakob et al., 2009). Despite progress-to-date, there is still a significant frontier to be explored in switchgrass given its high genetic diversity (Lemus et al., 2008; Casler, 2012). Conventional phenotyping studies have been implemented to identify, principally, high biomass phenotypes. However, these trials are performed manually, which is resource-intensive and requires destructive harvests. Also, the results obtained from manual evaluations are prone to assessment errors and are limited in time and space (Vogel et al., 2011).

Reliable and efficient automated high-throughput phenotyping of switchgrass, especially to predict end-of-season biomass, would be a significant advance in the field. Thus, the overriding goal is to rapidly collect high-quality data from a standoff for which current methods are not suited (Walter et al., 2019). One important automated phenotyping tool is light detection and ranging (LiDAR) technology. LiDAR is a laser-based sensor that produces high-throughput and high-density three-dimensional (3D) point clouds by photon-counting (Lim et al., 2003). Another tool that complements LiDAR is multispectral imaging, which collects vegetation spectral indices to be analyzed together with LiDAR data. LiDAR has been widely used for plant architecture measurements such as plant height (Bendig et al., 2015; Jimenez-Berni et al., 2018). While optical imagery models have been made to non-destructively estimate plant biomass (Hansen and Schjoerring, 2003; Bendig et al., 2014), these models have been criticized for the low accuracy and high uncertainties in estimating biomass (Shabanov et al., 2003; Garrigues et al., 2006). One problem inherent to optical imagery techniques is the potential for natural light saturation for detecting the high-density biomass plants (Mutanga and Skidmore, 2004; Li et al., 2014). Integration of LiDAR and spectral index technologies have been used to address these underlying factors determining plant biomass varying with plant type and phenotyping parameters (e.g., plant height and fractional canopy cover) (Tucker et al., 1985; Popescu et al., 2003; Li et al., 2018).

In order to apply current automated phenotyping technologies to estimate switchgrass biomass, our goal was to incorporate plant phenotyping parameters into the spectral index-based biomass models. Our testbed was a common garden in Knoxville, TN, United States growing a diverse collection of switchgrass clones (330 genotypes) under low and moderate nitrogen fertility conditions. The objectives of the present study were to (1) use standoff automation to measure plant height and perimeter for each plant from an over-the-field vertical perspective using unmanned aerial vehicle (UAV)-based LiDAR technology, (2) to improve the capacity of remote sensing to model plant biomass by integrating LiDAR and imagery technologies, and (3) to assess the stability of our biomass model over the growing season. To our knowledge, this is the first study to fully extend UAV technologies into the assessments for high-throughput switchgrass phenotyping and biomass yield estimating under field conditions.

## Materials and Methods

### Switchgrass Field Site and Experimental Design

**FIGURE 1:**
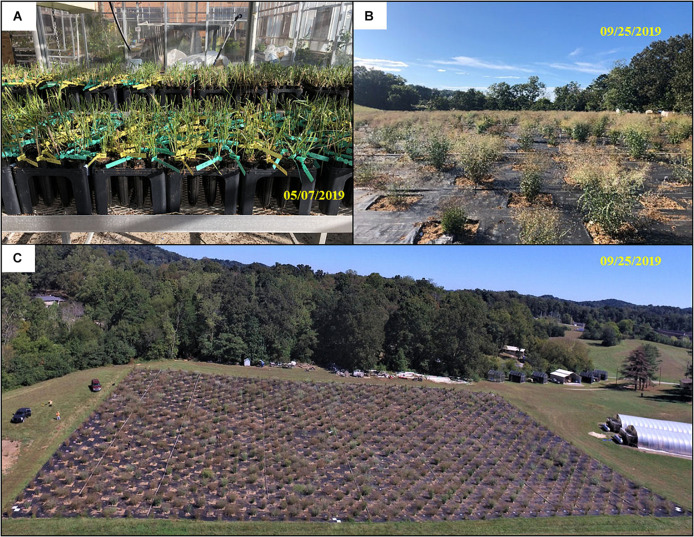
Switchgrass field establishment. **(A)** The 330 switchgrass accessions in pots awaiting transplanting to the field; **(B,C)** status of switchgrass growth nearly 4 months after transplantation into the field site.

The 75.2 × 122.5 m common garden was located at the University of Tennessee Plant Sciences Unit of the East Tennessee Research and Education Center (ETREC). The 330-switchgrass natural variant accessions were transplanted from a greenhouse to the field with four tillers per plant on May 28 and 29, 2019 (Figures 1A,B). The switchgrass clones used are mostly lowland (tetraploid) accessions provided by Dr. Thomas Juenger, University of Texas – Austin (Lowry et al., 2019). The field experiment is part of a switchgrass domestication project consisting of 330 accessions planted under two nitrogen (N) fertility treatments, one with moderate (135 kg of N ha^–1^) and another with low (0 kg of N ha^–1^) supplementation in July 2019. Each accession has four replicates in the field (2 replicates per N treatment), totaling 1,320 switchgrass plants, which were arranged in honeycomb design with ∼2.5 m interplant spacing (Figure 1C). The N treatment is part of another long-term study focusing on nitrogen use efficiency (NUE) in switchgrass. This provided the opportunity to determine the impacts of differential growth conditions on automated measurements. The experimental field was surrounded by switchgrass cv “Blackwell” border plants. The N treatment plots were separated by a centralized row of border plants. Water-permeable weed cloth coverage on the soil surface was used to reduce weed interference. Switchgrass was planted in 1 × 1 m holes in the cloth. Any weeds growing adjacent to switchgrass plants were manually removed.

### Manual Measurements of Plants

Each plant canopy perimeter and height was manually measured twice during the field season: once in August 2019 (mid-season) and once in December 2019 (end-of-season). The plant canopy height measurement consisted of the distance from ground level to the tip of the tallest central tiller using a tape. The plant canopy perimeter was determined with distance measurement for the outside border of plant canopy from a vertical viewpoint. Measurements were made without touching the plants, and required two people to work 2 days each time. The aboveground plant biomass was determined at the end-of-season after plant senescence (Table 1). Dry above ground biomass was determined at the end of the season by harvesting and weighing each plant. Subsequently, the ten tallest tillers were collected from each plant and oven-dried at 45°C for 72 h to determine the ratio of dry-to-fresh weight. Total dry biomass was determined by calculating the percentage of water loss recorded for each subsample and subsequently applying the water loss percentage to the respective total “wet” biomass weight for each plant. Plants with ten or fewer tillers were not subsampled, and whole plants were subjected to the same drying conditions and dry biomass was recorded for each plant. End-of-season biomass measurements required two people 2 weeks of work for harvesting, biomass drying, and recording of biomass by plant.

**TABLE 1:** UAV data routing observations and manual measurements in the growing season.

Date collection sate	Multispectral image	LiDAR	Manual measurements
08/14/2019	×		Plant perimeter and height
09/09/2019		×	
09/19/2019	×		
09/25/2019	×		
10/03/2019	×		
10/17/2019	×		
11/01/2019		×	
11/18/2019	×		
12/04/2019	×	×	Plant perimeter and height
01/21/2020	×	×	Plant biomass

### UAV Observations

**FIGURE 2:**
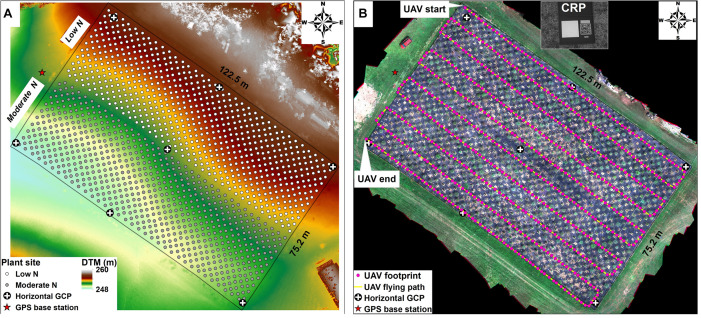
UAV ground control and flight operations. **(A)** Bare ground elevation data (i.e., digital terrestrial models, DTM) obtained after the switchgrass harvest (January 21, 2020) using UAV-based LiDAR scanning technology. The UAV ground control system included a GPS base station used for post-processing differential correction of LiDAR point clouds, horizontal GCP used for geometric rectification of multispectral image. Shown is the planting site for the 1,320 switchgrass plants. **(B)** UAV route over the field, as well as the calibrated reflectance panel (CRP) used to convert raw pixel values from multispectral images to absolute reflectance, where the CRP image was obtained before or after the flight.

Over the mid-to-late growing season we made 10 UAV flights to take single observations to estimate each trait by plant (Table 1) using a Matrice 600 UAV Pro model (DJI Inc., Shenzhen, China) equipped with multiple sensors including M200 Series Snoopy M8 LiDAR scanner (LiDARUSA Inc., Hartselle, AL, United States), and Red Edge-MX camera (MicaSense, Inc., Seattle, WA, United States) and strict ground control (Figure 2A). Flights were performed on cloud-free days between 10:00 am and 12:00 pm with an automatic mode using the drone flight planning mobile app – Pix4Dcapture (Pix4D Inc., Prilly, Switzerland) at 20 m above the ground and speed of approximately 4 km per hour (Figure 2B). The settings of image coverage overlapping between UAV-footprint snapshots was 85% in front and 70% on sides. The UAV-footprint shooting images over the field (Figure 2B) at a sampling resolution of 1 × 1 cm were mosaicked and transformed into the absolute reflectance images along with the image of the calibrated reflectance panel (CRP) captured prior to implementing flight mission, including blue (475 nm), green (560 nm), red (668 nm), red-edge (717 nm), and near-infrared (842 nm) bands, using Pix4Dmapper (Pix4D Inc., Prilly, Switzerland). Afterward, geometric rectification for the multispectral image was manually performed using the georeferencing tool in ArcGIS software (Esri Inc., Redlands, CA, United States) according to seven ground control points (GCP), which were evenly preassigned over the field and accurately measured using the global positioning system (GPS) base-station with a <4 cm horizontal accuracy. The LiDAR data obtaining was operated by tracking distances and angles through eight individual lasers at a shooting frequency of 440,000 points/s, along with the sensor position (i.e., latitude, longitude, and altitude) through the Global Navigation Satellite Systems (GNSS) and the sensor orientation (i.e., pitch, roll, and yaw) through the inertial measurement unit (IMU), as well as the real-time GPS base-station recording. To achieve a highly precise positioning for both horizontal and vertical (±3 cm), the raw LiDAR data recorded by those devices were repositioned by post-processing differential corrections based on the GPS base-station as well as the IMU data using Inertial Explorer Xpress 8.7 (NovAtel Inc., Calgary, AB, Canada) and were then further converted into the point clouds in the LAS (*.las*) format using ScanLook Point Cloud Creation (LiDARUSA Inc., Hartselle, AL, United States).

### Automated Phenotyping Measurements

**FIGURE 3:**
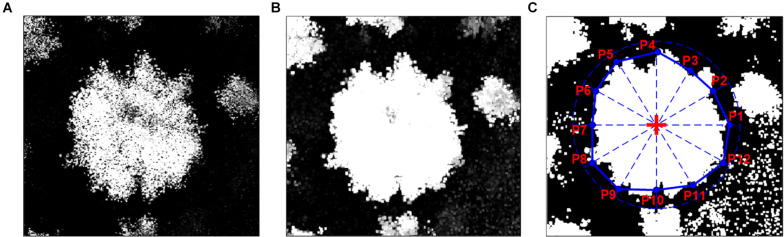
Process of plant phenotyping parameter extraction. **(A)** The gridded plant height. **(B)** The spatially filtered plant height. **(C)** The binarization of plant height for identifying the location of plant canopy based on the central coordinates of plants (red + symbol) and the 2 m scanning line following the 30-degree interval, where the blue points (i.e., P1, P2, …, to P12) are the location of intersection between plant canopy boundary and scanning line.

The 3D plant canopy was delineated by the point clouds that were composed of a high-density mass of point vectors, with each one having its own set of horizontal positioning (latitude and longitude), elevation coordinates, and additional attributes. Individual plant canopy polygons were identified using the MATLAB programming package (Math Works Inc., Natick, MA, United States) through three steps, including plant height calculation, spatial filtering, and boundary identifying (Figure 3).

(1) Plant height calculation: Individual.*las* files were combined into LAS datasets (.*lasd*) that were further interpolated into 1 × 1 cm gridded digital surface models (DSM, generated during the growing season and representing the incorporation of the bare ground elevation and plant canopy) in the Tagged Image Format File (*.tiff*) format using ArcGIS software to match the sampling results for the multispectral image. To precisely calculate plant canopy height, the bare ground elevation data, namely the digital terrestrial models (DTM), were generated by UAV-based LiDAR scanning technology after the switchgrass harvest (Figure 2A). The plant canopy height models (CHM) were calculated by DSM in the growing season, subtracting the DTM (e.g., Figure 3A).
(2) Spatial filtering: Generally, the plant canopy contains gaps between leaves that impact the complete identification of plant canopy. To simplify the process, we applied a spatial filter to the CHM to fill the gaps to unite all the pieces of canopy together. Specifically, the order-statistic filtering function (i.e., *ordfilt2*) with the domain of 5 × 5 pixels and the value of the 25th percentile was used to smooth the CHM (e.g., Figure 3B).
(3) Boundary identifying: The CHM was binarized with the threshold of 10 cm, below which was considered as the invalid value resulting from point cloud positioning error as well as ground relative elevation changes. Based on the central coordinates of the plant, the gridded plant canopy was divided from CHM, and the function of *bwboundaries* was used to trace the exterior boundary of the plant canopy. To simplify the boundary, a 2 m line that originated from the center of the plant was used to detect the intersected points between initial plant canopy boundary and scanning line following an interval of 30-degree. Generally, 3–12 points were identified, depending on the overlapping case with the surrounding plants (e.g., Figure 3C). The identified points were further converted into the polygon in the Esri shapefile (*.shp*) format using *shapewrite* function.

After obtaining the plant canopy polygon for each plant, plant canopy perimeter was measure in a similar way to manual measurements by calculating the distance around the outside plant canopy border as viewed from a vertical perspective. Plant canopy perimeter and area were calculated using the functions of *perimeter* and *polyarea*, respectively. By overlaying each plant canopy polygon to the gridded CHM, the maximum CHM value was identified and used for comparison with manual ground-truth measurements that were implemented referring to the top of the central panicles in the plant, and the mean CHM value over the plant canopy was used for the subsequent driving of the UAV-biomass model. The mean reflectance for each plant and band in the multispectral image was calculated to derive the spectral vegetation index as another driving variable for UAV-biomass modeling.

### Plant Biomass Model and Evaluation

The plant canopy perimeter and height, as vital measurements for plant phenotyping structure characteristics, are theoretically related to the magnitude of plant stems (Fernandez et al., 2009). Also, the spectral index was developed based on the fact that leaf chlorophyll electromagnetic spectra measurements are highly correlated with plant leaf density (i.e., leaf area index – LAI) (Broge and Leblanc, 2001). Here, we modeled the plant biomass as a linear combination of phenotyping measurements and spectral index response in the form of (Eq. 1):

(1) $$f_{\text{Biomass}}=\left(m\times \text{SI}\right)\times \text{CP}\times \text{CH}$$

where CP and CH refer to the plant canopy perimeter and height, respectively. These phenotyping variables change significantly during the growing stage, but are supposed to approach to a constant status after peak growing season; SI is the spectral index calculated from UAV-based reflectance bands; *m* is a SI-sensitive coefficient relying on a specific spectral index as well as plant phenological stage. To evaluate biomass yield for the mature plants, all driving variables were obtained during the peak growing season to assure a robust prediction with the UAV-biomass model. This is imperative given plants may “de-green” with plant senescing after peak growing season, and “de-greening” may lower the performance of spectral index (Tillack et al., 2014). Several widely used indices were explored for SI including the spectral index developed in the early period, such as the ratio vegetation index (RVI; Eq. 2) (Pearson and Miller, 1972) and the normalized difference vegetation index (NDVI; Eq. 3) (Rouse et al., 1974), as well as spectral index suggested later for improving sensitivity to vegetation, such as the enhanced vegetation index (EVI; Eq. 4) (Huete et al., 1997) and the normalized difference red edge index (NDRE; Eq. 5) (Hansen and Schjoerring, 2003). These indices were calculated using the following equations:

(2) $$\text{RVI}=\frac{R_{N\,I\,R}}{R_{R\,e\,d}}$$

(3) $$\text{NDVI}=\frac{R_{N\,I\,R}-R_{R\,e\,d}}{R_{N\,I\,R}+R_{R\,e\,d}}$$

(4) $$\text{EVI}=2.5\times \frac{R_{N\,I\,R}-R_{R\,e\,d}}{R_{N\,I\,R}+6\,R_{R\,e\,d}-7.5\,R_{B\,l\,u\,e}+1}$$

(5) $$\text{NDRE}=\frac{R_{N\,I\,R}-R_{R\,E}}{R_{N\,I\,R}+R_{R\,E}}$$

where *R*_*NIR*_ is the reflectance at the near-infrared wavelength, *R*_*Red*_ is the reflectance at the red wavelength, *R*_*Blue*_ is the reflectance at the blue wavelength, and *R*_*RE*_ is the reflectance at the red-edge wavelength.

Standard criteria, namely the Pearson coefficient (*r*), root mean square error (*rmse*), and relative error (*re*), were used to evaluate how well the assembly of phenotyping measurements and SI-response predicted the biomass compared to manual measurements.

## Results

### UAV-Based Plant Phenotyping Parameters and Validations

**FIGURE 4:**
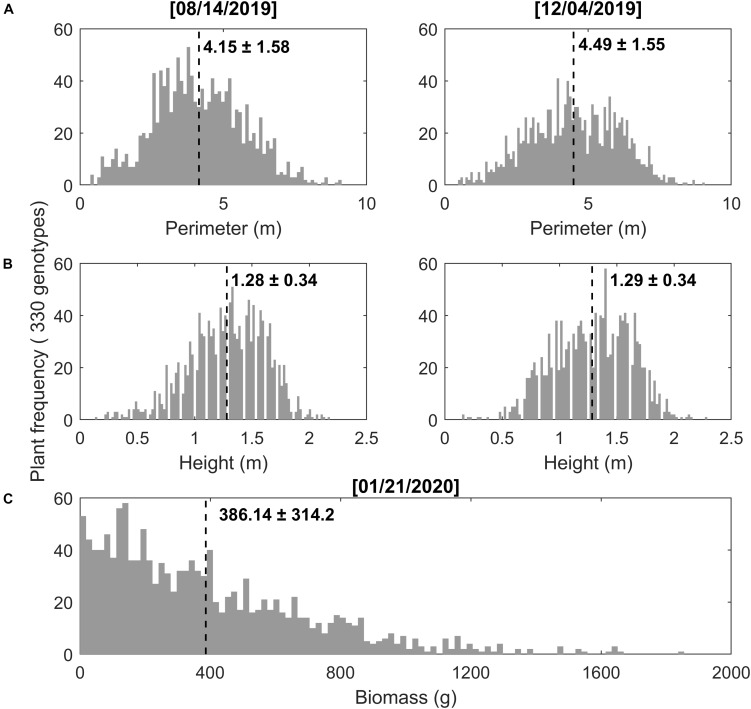
Manual phenotyping measurements for 330 genotypes of switchgrass during mid-season (August 14, 2019) and end-of-season (December 04, 2019), as well as biomass harvest (January 21, 2020). **(A)** Plant perimeter, **(B)** plant height, and **(C)** plant biomass, where the numbers on the figure represent the mean and standard deviation (std) for 1,320 plants with 330 genotypes and four repetitions for each genotype.

There was a wide range of values from manual measurements of switchgrass perimeter, height, and biomass yield among the 330 genotypes (Figure 4). Plant perimeter ranged from 0.36 to 12.37 m with an average (and standard deviation) of 4.15 m (±1.58) and 4.49 m (±1.55) for measurements taken at the mid- and the end-of-season, respectively (Figure 4A). In the same way, switchgrass height (i.e., central panicle) ranged from 0.13 to 2.29 m, with an average of 1.28 m (±0.34) and 1.29 (±0.34) (Figure 4B). Dry biomass ranged from 2 to 1,855 g per plant, with an average of 386.14 g (±314.2) (Figure 4C). Trait variation may be related to genetic diversity among the accessions (Martinez-Reyna and Vogel, 2002; Casler, 2012).

**FIGURE 5:**
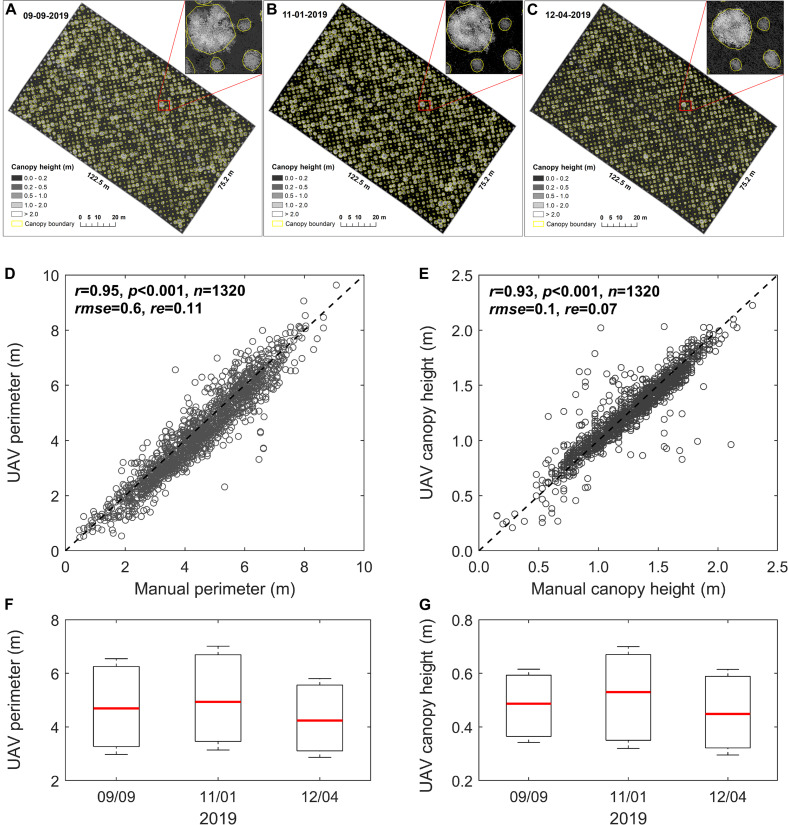
Changes in UAV-based plant phenotyping parameters and validation. **(A–C)** Spatiotemporal changes in plant canopy perimeter and height based on UAV measurements during the growing season; **(D,E)** validation of the UAV-based plant perimeter and height with manual measurements for a total of 1,320 plants, where plant canopy height was compared based on the top of the central panicles. The UAV and manual measurements were collected on December 4, 2019; **(F)** and **(G)** boxplots of the changes in plant perimeter and height, respectively, from peak season to the end of the season, where the red line on the box indicates the median, and the bottom and top edges of the box indicate the 25^*th*^ and 75^*th*^ percentiles, respectively.

We applied the programming process (involving three steps, i.e., plant height calculation, spatial filtering, and boundary identifying) to the LiDAR point clouds collected from early peak season (Figure 5A) to the end-of-season (Figures 5B,C) for determining switchgrass phenotyping parameters including plant canopy height and perimeter. Compared to the manual measurements (i.e., from December 4, 2019), we achieved promising results for plant canopy perimeter (*r* = 0.95, *rmse* = 0.6, and *re* = 0.11; Figure 5D) as well as canopy height (*r* = 0.93, *rmse* = 0.1, and *re* = 0.07; Figure 5E) using LiDAR. The box statistics showed that plant phenotyping parameters for both perimeter and height slightly increased from September 9 to November 1, 2019 (Figures 5F,G). Afterward, a notable decrease in height was observed on December 4, 2019. This result might be attributed to plant lodging responses associated with genetic characteristics of each genotype, as well as interactions with environmental effects such as rainfall and snow (Tripathi et al., 2003).

### Performance of UAV-Biomass Model

**FIGURE 6:**
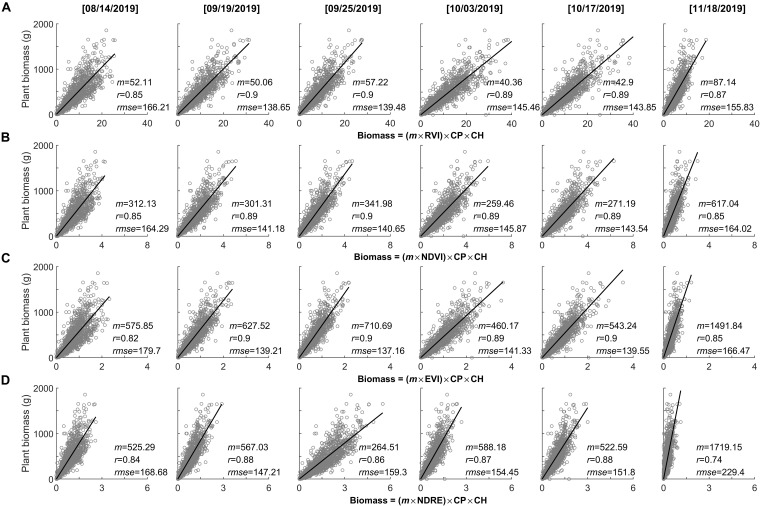
Performance of the assembly of phenotyping measurements (CP, canopy perimeter; CH, canopy height) and spectral index in predicting the plant biomass with changes of the growing season, as well as a different spectral index. **(A)** RVI, **(B)** NDVI, **(C)** EVI, and **(D)** NDRE.

Using the maximum plant phenotyping parameters (i.e., plant perimeter and height on November 1, 2019; Figure 5) as a static forcing variable, we explored the UAV-based biomass models accompanied with changes of varying spectral index and phenological process from early peak season (i.e., August 14, 2019) to the end-of-season (i.e., November 18, 2019) (Figure 6). We found that the assembly of phenotyping measurements (plant height and perimeter) and spectral index demonstrated promising performance in predicting the plant biomass (*r* ≥ 0.74), but also varied among the spectral indices as well as phenological stages (*r* = 0.74–0.9). Noticeably, the spectral indices derived from peak season to just prior to the end-of-season (e.g., September 19 to October 17, 2019) demonstrated consistent and robust performance in predicting plant biomass (*r* ≥ 0.86). Compared to NDRE, the commonly used spectral indices of RVI, NDVI, and EVI demonstrated a stronger relationship with plant biomass (*r* ≥ 0.89). Out of these three spectral indices, EVI demonstrated the lowest estimated bias (*rmse* ≤ 137.16). In contrast, using the spectral indices derived from the early peak season (i.e., August 14, 2019) and the end-of-season (i.e., November 18, 2019) were weaker predictions of plant biomass (*r* ≤ 0.87).

### Plant Responses to N Treatments

**FIGURE 7:**
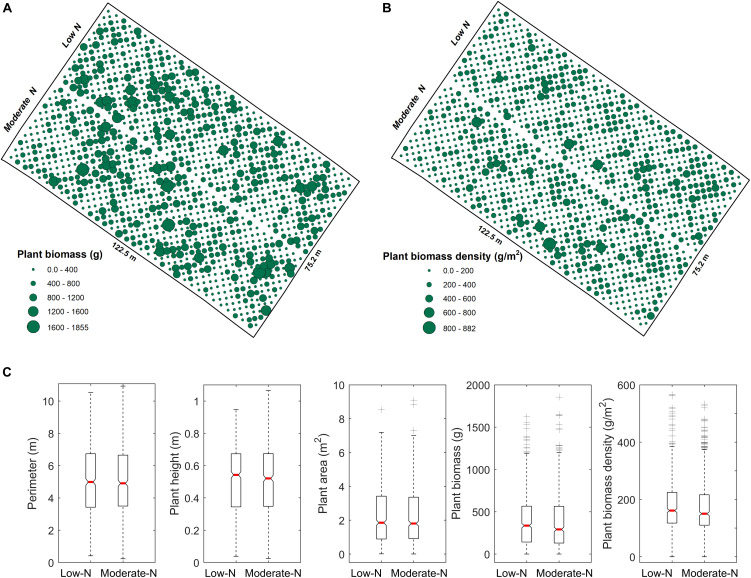
Comparisons of different N treatments for switchgrass. **(A)** Spatial distribution of switchgrass biomass over the experimental field mapped to individual plants. **(B)** Spatial distribution of switchgrass biomass density that is normalized by plant canopy coverage area. **(C)** Boxplots for plant phenotyping parameters (i.e., plant perimeter, height, and plant area), as well as plant yield (single plant biomass and biomass-density) between two levels of N treatments, where the red line on the box indicates the median, and the bottom and top edges of the box indicate the 25^*th*^ and 75^*th*^ percentiles, respectively.

**FIGURE 8:**
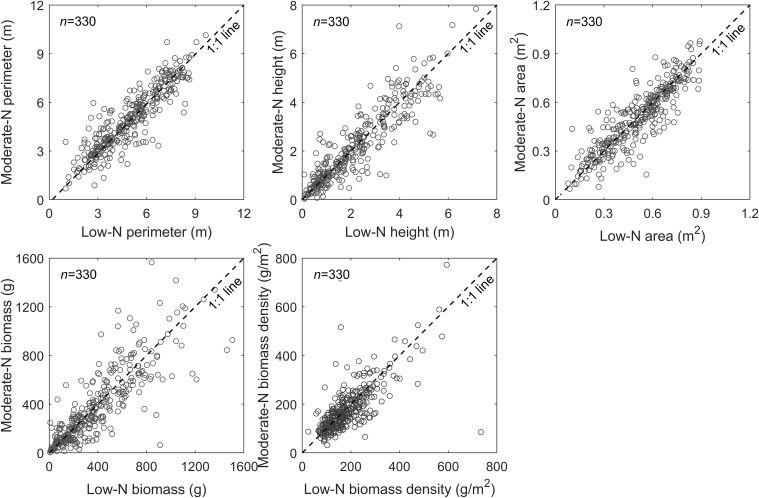
Comparisons of two contrasting N treatments for each genotype with four replicates (2 replicates per N treatment). The comparisons are performed among the plant canopy perimeter, height, and area, as well as single plant biomass and biomass-density, where the data for the plant canopy perimeter, height, and area are derived from peak season of plant growth (i.e., September 25, 2019). Each data point represents an average of two replicates per each N treatment (low and moderate) for a total of 330 switchgrass genotypes.

Manual and automated measurements of the variables (e.g., plant height, perimeter, area, biomass density, and biomass production) demonstrated there were no significant differences in switchgrass growth between the low and moderate N treatments (Figure 7). These results were strongly correlated between the automated and manual methods (Figure 5), and we found the differential N growth conditions had no effect on automated phenotypic characterization. Based on UAV measurements, we found similar patterns in the distribution of switchgrass plant biomass (Figure 7A), as well as biomass density over the field (Figure 7B) between the low and moderate N fertilization plots. In contrast, we observed a general positive plant growth response to the N fertilization over the 330 switchgrass genotypes (Figure 8). These observations suggested that the high genetic variability of the 330 genotypes is responsible for the large-ranging differences in plant growth factors rather than the N fertilization itself (Cassida et al., 2005). However, there were patterns among genotypic responses to N treatments. We characterized genotype responses as: a) N-positive responsive genotypes, in which growth was positively associated with N (Figure 8, representative genotypes above 1:1 line); b) N-neutral genotypes that had congruent growth in both N treatments (Figure 8, representative genotypes at 1:1 line); and c) N-negative genotypes that had lower growth with more N (Figure 8, representative genotypes below the 1:1 line).

## Discussion

### LiDAR-Based Plant Phenotyping Measurements

Unmanned aerial vehicle-based LiDAR scanning technology was very useful in measuring switchgrass plant morphological traits over the field. The automated method we are reporting in the present study was validated by the manual measurements with a wide range of phenotypic variabilities. Our assessment demonstrates the reliability of the system for use in different switchgrass growing conditions with high accuracy. It should be noted that the LiDAR sensor used in this study (i.e., M200 Series Snoopy M8 LiDAR scanner) can only record the single echo, implying there may be some uncertainty in accurately calculating plant canopy height (i.e., CHM) relying on single-pass-obtained point cloud data (James and Robson, 2014). To simplify the processing procedure and ensure the measuring accuracy, plant canopy height in the growing season was determined by DSM generated in the plant growing season by subtracting DTM (Figure 2A) generated after all the plants are harvested (i.e., CHM = DSM − DTM). This strategy could be applicable to this study by assuming that the changes in the background surface elevation are negligible. However, most situations require capacities of large-scale detection and timely variable calculation. In these cases, the full waveform or multi-echo LiDAR-scanning technologies can be helpful for producing DTM, DSM, and CHM variables at once through the algorithm of decomposing LiDAR waveforms (Reitberger et al., 2008; Mallet and Bretar, 2009). The value of LiDAR scanning technology is not only characterized by its highly efficient reproducibility and accuracy (Madec et al., 2017) but also due to its irreplaceability. For example, we conducted manual measurements for plant canopy height by sampling representative tillers on each plant. However, because there was a considerable height variation among tillers, the single or multiple tiller height measurements using manual methods, e.g., tape measure will inevitably produce uncertainty in delineation of plant height. Instead, the highly dense LiDAR point clouds have higher repeatability to delineate the height variations for plant tillers, and that consequently can ensure the robust phenotyping measurements, as well as the precise yield prediction with UAV-biomass model. For example, when manual height measurements were made, it took two people 2 days to measure the tallest tiller for each of the 1,320 plants. The single point measurement for each plant may not be an absolute representation of “true plant height,” whereas the UAV platform is scalable and able to collect a data cloud for each plant.

We explored the applicability of structure-from-motion (SFM) algorithms using the Pix4Dmapper programming package for the generation of the DSM and DTM based on a large set of overlapping images (Oliensis, 2000). When compared with the LiDAR method, we found that SFM method was suboptimal to capture plant canopy structural details. Lussem et al. (2019) showed that SFM-derived CHM provided a varying performance in predicting grassland biomass, indicating this method may not be widely adaptable. The success of SFM depends on several factors, including the complexity of research object, UAV flight control accuracy, image quality, as well as the selection of SFM algorithms (Dandois and Ellis, 2013; Remondino et al., 2014). Based on our study, we suggest that SFM may not be optimal for quantifying small objects with a high degree of accuracy. Rather, SFM may be useful to 3D visualization or structure parameter measurements specific to large objects such as trees and buildings (Bolles et al., 1987). In addition, we experimented with extracting plant canopy perimeters through the spectral index (e.g., NDVI) calculated by the multispectral image. Once a threshold used for segmenting spectral index image is determined, we found this method is applicable for the calculation of plant canopy coverage perimeter and area. However, one substantial problem is determining the appropriate threshold value, which varies with dynamic leaf chlorophyll content during plant development. For example, after switchgrass growth peaks in late summer and senescence ensues, the leaves will be less green. An undistinguished phenotype may be observed along with diverse stresses (e.g., drought, plant pests, and diseases) over the growth season (Anjum et al., 2011; Mahlein et al., 2013). In contrast to the optical image processing method, the LiDAR scanning method appeared to be more robust and applicable for estimating switchgrass phenotypic parameters, such as plant canopy height and perimeter.

### Flexibility of UAV-Biomass Model

In recent years, UAV-based biomass models have been developed using UAV platforms equipped with a LiDAR scanner and/or multispectral sensor. When the LiDAR scanning is used, plant biomass is modeled as the function of CHM, such as [α^∗^e^(β^*CHM)^] (Bendig et al., 2014). CHM models have high predictive value because the technique precisely delineates plant stem density and height, but suboptimally estimates biomass density in the unit of volume (Asner et al., 2012). Some biomass estimation models largely ignore image spectral index with forms such as [α^∗^SI + β] or [α^∗^SI^β^] (Bendig et al., 2015). However, because of the attenuation of electromagnetic wave propagation when passing through a very dense vegetation canopy, namely the saturation of optical remote sensing, these kinds of models may not be appropriate to predict biomass (Shabanov et al., 2003; Mutanga and Skidmore, 2004; Li et al., 2014). Meanwhile, the model coefficients (i.e., α and β vary with choice of spectral index, and its associated phenological stage, as well as taxa. These variations in model forms and coefficients prevent us from cross-analysis among traits, including phenotyping heterogeneity, biomass composition and density, as well as evaluating NUE (Hardin et al., 2013; Li et al., 2018). Taking this a step further, it will impede our understanding of whether biomass production is largely explained by genotype and canalized phenotypes (Casler, 2012). In the present study, a standardized model is proposed to estimate switchgrass biomass and its coefficients. The model was relatively straightforwardly and applicable to efforts to improve switchgrass cultivation as a bioenergy feedstock across diverse environmental conditions.

**FIGURE 9:**
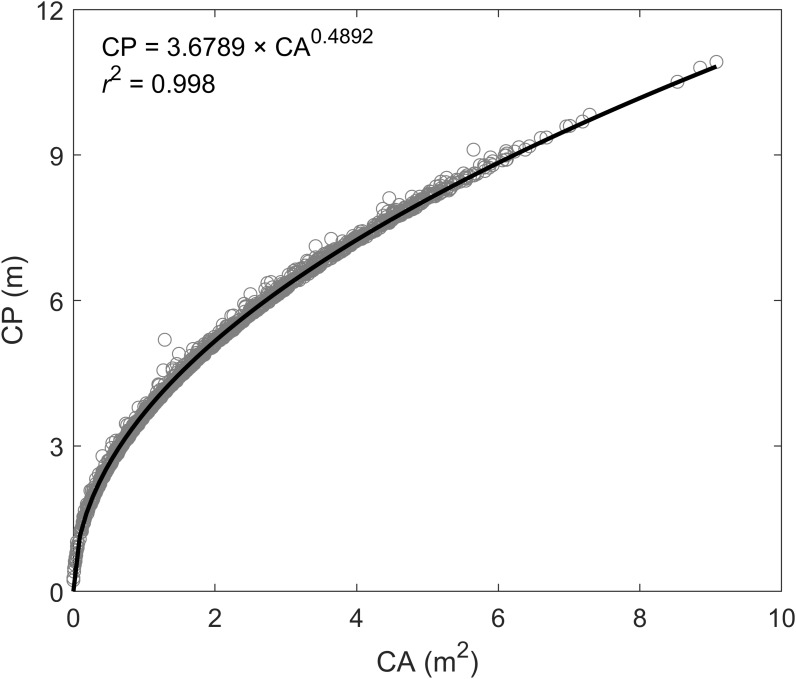
The functional relationship between plant canopy area (CA) and canopy perimeter (CP) according to the UAV-based phenotyping measurements.

UAV-based remote sensing technologies are of value not only for increasing precision of trait measurement (e.g., biomass and LAI) but also for superior performance of mapping large-scale vegetation coverage areas (CAs) (Baret and Guyot, 1991; Jimenez-Berni et al., 2018). Accordingly, when the UAV-based biomass model proposed here is applied to plants grown under agronomic conditions with broad spatial and temporal scales, it is important to validate methods. First, plants grown under agronomic conditions vary in a continuous or undistinguished pattern, along with a certain fractional soil exposure. In this case, instead of using the purely individual plant canopy parameters (i.e., plant phenotyping parameters and spectral index) for modeling biomass, UAV-based remote sensing images for each pixel cell at a certain spatial resolution (e.g., 0.5 × 0.5 m, mostly relying on UAV flying altitude) may be used to build models. To reduce the impacts of bare soil on the spectral index, we suggest using the pure vegetation index (PVI) proposed by Li et al. (2016) for substitution of the spectral index in the UAV-biomass model. Since the soil component is completely excluded from PVI, based on the spectral mixture analysis (SMA) method (Adams et al., 1995), this substitution can be congruent with the role of pure vegetation canopy spectral index used in this study. It will hold a proportional response to changing biomass values. As for plant perimeter, initially, we thought that the assembly of plant canopy area and height could be mathematically used to determine the plant canopy volume magnitude, while adding spectral index can play a role in qualifying the biomass density of the canopy volume. However, we found that this type of assembly suffers a non-proportional response to biomass changes, resulting in a higher bias in predicting biomass yield. The choice of using the different model forms (e.g., exponential or polynomial forms) is possible, but may complicate model applications, given the variations of model form and its coefficients. In addition, we found that plant perimeter plays an important role in the optimization of modeling plant biomass yield. However, plant perimeter used here is only derived from each individual plant. As for biomass modeling based on a pixel cell, we suggest using a highly related function for converting from plant canopy CA in the pixel cell to plant perimeter, which is developed based on UAV phenotyping measurements in the switchgrass field (i.e., CP = 3.6789 × CA^0.4892^, *r*^2^ = 0.998; Figure 9). Indeed, in agronomic fields of switchgrass, taking individual plant measurements, e.g., perimeter, will be challenging.

### Spectral Index Sensitivity

**FIGURE 10:**
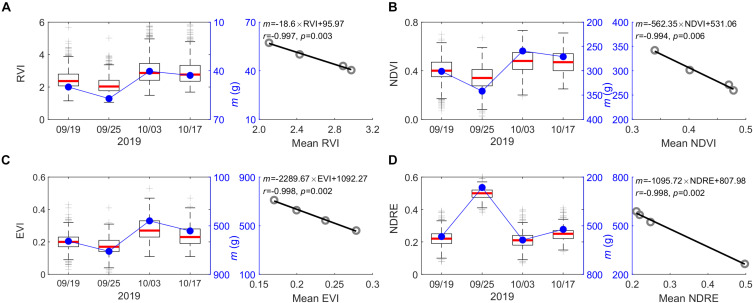
Relationship between the coefficient of *m* in UAV-biomass models and plant phenotype changes qualified by the various spectral indices from the early peak season to the end of the growing season. **(A)** RVI; **(B)** NDVI; **(C)** EVI; and **(D)** NDRE. Time-specific variations of the spectral index for the 1,320 switchgrass plants in the field are reflected by boxplot statistics, where the red line on the box indicates the median, and the bottom and top edges of the box indicate the 25^*th*^ and 75^*th*^ percentiles, respectively.

In some cases, after peak growth season, plants may “de-green,” which will alter the performance of spectral index used for plant monitoring (Tillack et al., 2014). However, based on varying spectral indices from peak season to before end-of-season (e.g., September 19 to October 17, 2019) used for the UAV-biomass modeling, we found an insignificant impact of plant phenological changes on its performance in modeling biomass (Figure 6). The only changes appear in the coefficient of *m* in the UAV-biomass model (i.e., Eq. 1), which varies with the choice of the spectral index (i.e., RVI, NDVI, EVI, and NDRE) and its association with the plant-phenological stage changing (Figure 10). This finding suggests that we have a broader time window (e.g., during September and October in this study) to reliably estimate the end-of-season plant biomass using UAV-based remote sensing technologies, rather than rushing into the peak growing season for UAV data collection; this stage would have maximum content of chlorophyll in the leaf. The *m* coefficient, calculated as the ratio between biomass yield and the assembly of plant phenotyping parameters and spectral index, i.e., [*m* = biomass/(SI × CP × CH)], represents the change in biomass yield per unit change in the integration of plant multi-traits. These traits vary between genotypes and among plant species, as well as phenological stages. Generally, *m* is determined through *in situ* destructive measurements along with UAV data collecting (Walter et al., 2019). By exploring the time series spectral index, we found that the *m* magnitude is significantly positively correlated with the spectral index changes that are associated with plant phenological stages (*r* = 0.994–0.998, *p* ≤ 0.006; Figure 10). Among the selected spectral indices, the highly sensitive spectral index of EVI demonstrated a more robust performance to determine the *m*-value for calibration of the UAV-biomass model (i.e., *m* = −2289.67 × EVI + 1092.27; *r* = −0.998, *p* = 0.002; Figure 10C). This finding implies that the coefficient of *m* in the UAV-biomass model can be determined according to the spectral index of its property, rather than through *in situ* destructive sampling measurement of plant biomass (Li et al., 2018), which is not desirable.

**FIGURE 11:**
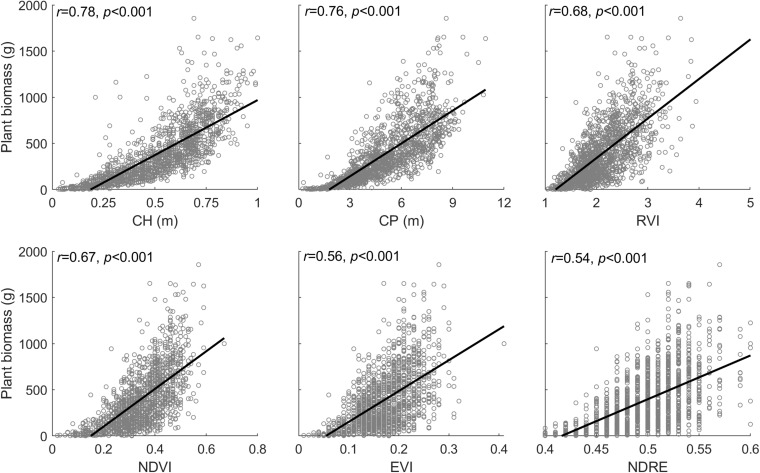
Relationship between the plant phenotyping parameters (CH, canopy height; CP, canopy perimeter), spectral index (i.e., RVI, NDVI, EVI, and NDRE), and plant biomass, where the corresponding data are derived from peak season of plant growth (i.e., September 25, 2019).

Unmanned aerial vehicle-biomass models varying with diverse forms are primarily attributed to a non-linear response to biomass increasing changes, which is so-called the natural saturation of optical remote sensing detections (Baret and Guyot, 1991; Gitelson, 2004). Based on the experiments from this study, we found that the plant phenotyping variables (e.g., plant canopy height and perimeter) measured by LiDAR technology, and spectral index measured by multispectral image all are subjected to the influence of saturation with varying degrees in response to increasing biomass in the plant canopy of leaves and stems (Figure 11). Overall, out of these input variables, plant canopy height is the single best trait to estimate end-of-season aboveground biomass (*r* = 0.78, *p* < 0.001), followed by plant perimeter (*r* = 0.76, *p* < 0.001), and then diverse spectral indices (*r* = 0.54–0.68, *p* < 0.001). Among the spectral indices, a slight difference exists when the individual spectral index is used for biomass modeling, but the insignificant difference is found when assembled with plant canopy perimeter and height.

### Effects of N Fertilization on Switchgrass Growth

Nitrogen is an essential nutrient that is important to manage in bioenergy and forage crop production (Monti et al., 2019). We observed that N-supplementation had no significant effects on the global biomass production of the 330 switchgrass genotypes included in this study. This is in contrast to other studies that have shown switchgrass is more productive under N-fertilizer treatments when water is not limited (Schmer et al., 2012; Emery et al., 2020), and when N-supplementation is applied to established fields and during more than one year (Jung and Lal, 2011). However, N-supplementation does not always result in higher biomass production and may have unintended effects on switchgrass growth (Emery et al., 2020). The present study was performed during the establishment year (year one), where switchgrass establishment has been reported to be slow and yield reduction has been observed in the first year (Baxter et al., 2014). This factor may have had a negative influence on biomass production obviating any potential positive effects of N supplementation. However, we emphasize the contribution of N uptake in switchgrass still needs further investigation, especially with the support of UAV-based multi-trait measurements proposed in this study. Meanwhile, to elucidate underlying mechanisms in switchgrass NUE from the perspective of genetic characteristics will be a concern-deserved topic in the follow-on study.

## Conclusion

Unmanned aerial vehicle (UAV)-based LiDAR and multispectral technologies were assessed for their application of high-throughput phenotyping of switchgrass and biomass estimation in the field. We found that UAV-based LiDAR is a useful tool for the precise qualification of plant phenotypic indicators (i.e., plant canopy perimeter, and height). Furthermore, a relatively simple and standardized model was developed for the estimation of switchgrass biomass yield through combing plant phenotyping characteristics (e.g., plant canopy height and perimeter) measured by LiDAR technology, and plant biomass density, which is detected by a widely used spectral vegetation index. We found that combining these phenotypic indicators significantly improves the performance of the spectral index in modeling and estimating biomass yield in a non-destructive manner. Finally, we found that, globally, N fertilization had non-significant effect on switchgrass phenotyping traits including biomass. In summary, the UAV-based approaches proposed in this study, including plant phenotyping automatic extracting method and biomass predicting model, facilitated high-throughput and precise phenotype mapping, which should have impact on accelerating bioenergy crop breeding as well as practical use in the field to estimate switchgrass biomass prior to destructive harvests at the end of the season.

## Data Availability Statement

The raw data supporting the conclusions of this article will be made available by the authors, without undue reservation.

## Author Contributions

FL and CP designed the study and performed the experiments and the result analysis. BW contributed to the manual measurements and field maintenance. CP, FL, and BW collected UAV and manual data. FL analyzed the UAV-based image data. CP analyzed the manual-based data. RM and MM provided critical insights into the method design and result interpretation. CS organized the study and revised the manuscript. All authors contributed to the text, and approved the final manuscript.

## Conflict of Interest

The authors declare that the research was conducted in the absence of any commercial or financial relationships that could be construed as a potential conflict of interest.
